# Analysis of *Clonostachys rosea*-Induced Resistance to Tomato Gray Mold Disease in Tomato Leaves

**DOI:** 10.1371/journal.pone.0102690

**Published:** 2014-07-25

**Authors:** Liana Dalcantara Ongouya Mouekouba, Lili Zhang, Xin Guan, Xiuling Chen, Hongyu Chen, Jian Zhang, Junfeng Zhang, Jingfu Li, Yijun Yang, Aoxue Wang

**Affiliations:** 1 School of Municipal and Environmental Engineering, Harbin Institute of Technology, Harbin, Heilongjiang, PR China; 2 College of Horticulture, Northeast Agricultural University, Harbin, Heilongjiang, PR China; 3 College of Life Sciences, Northeast Agricultural University, Harbin, Heilongjiang, PR China; 4 Alberta Innovates-Technology Futures, Vegreville, Alberta, Canada; 5 Department of Laboratory Medicine and Pathobiology, University of Toronto, Toronto, Canada; National Taiwan University, Taiwan

## Abstract

Tomato gray mold disease, caused by *Botrytis cinerea*, is a serious disease in tomato. *Clonostachys rosea* is an antagonistic microorganism to *B. cinerea*. To investigate the induced resistance mechanism of *C. rosea*, we examined the effects of these microorganisms on tomato leaves, along with changes in the activities of three defense enzymes (PAL, PPO, GST), second messengers (NO, H_2_O_2_, O_2_^−^) and phytohormones (IAA, ABA, GA_3_, ZT, MeJA, SA and C_2_H_4_). Compared to the control, all treatments induced higher levels of PAL, PPO and GST activity in tomato leaves and increased NO, SA and GA_3_ levels. The expression of WRKY and MAPK, two important transcription factors in plant disease resistance, was upregulated in *C. rosea-* and *C. rosea* plus *B. cinerea*-treated samples. Two-dimensional gel electrophoresis analysis showed that two abundant proteins were present in the *C. rosea* plus *B. cinerea*-treated samples but not in the other samples. These proteins were determined (by mass spectrum analysis) to be LEXYL2 (β-xylosidase) and ATP synthase CF1 alpha subunit. Therefore, *C. rosea* plus *B. cinerea* treatment induces gray mold resistance in tomato. This study provides a basis for elucidating the mechanism of *C. rosea* as a biocontrol agent.

## Introduction

The cultivated tomato *Solanum lycopersicum* is an herbaceous vegetable plant that belongs to the *Solanaceae* family. The tomato plant thrives at almost all latitudes. However, tomato crops may be susceptible to damage due to pests (insects, mites, nematodes and so on), fungal, bacterial or viral diseases, competition from weeds and vegetation accidents or abiotic stress.

Tomato gray mold disease, caused by *Botrytis cinerea*, is the serious disease that threatens tomato production in both the greenhouse and field. This disease affects not only tomato but also many other commercially important crops, such as grape, apple, pear, cherry, strawberry, kiwi, eggplant, carrot, lettuce, cucumber and pepper, which are grown either in the greenhouse or in the field. This fungus infects plants primarily through scratches on the plant surface, at it is also able to infect plants by penetrating healthy plant tissues. *B. cinerea* fungus secretes a large number of cell wall degrading enzymes (CWDEs) during the infection process, which explains why this fungus can penetrate the surfaces of healthy plants [1], [2].

Plant diseases can be controlled using synthetic fungicides, but the use of fungicides has been restricted due to their carcinogenicity, teratogenicity, high and acute residual toxicity, long degradation period, effects on environmental pollution and possible effects on human health due to direct consumption [3]. While resistant cultivars can be produced by breeding, no gray mold-resistant tomato materials have been produced to date. Therefore, new alternatives have been explored to reduce the use of synthetic fungicides. The use of biological measures to control this disease has become an inevitable pursuit in disease prevention and treatment, especially in the agricultural production process, through the development and use of microorganisms antagonistic to *Botrytis cinerea*.

The mycoparasite *Clonostachys rosea* has been tested successfully as a biological control agent against divergent fungal plant pathogens [4]. *C. rosea* is an antagonistic fungal plant pathogen that is widely present in soil and can produce a series of antibacterial metabolites. Many isolates of *C. rosea* are highly efficient antagonists against several plant pathogenic fungi, and studies have shown that this fungus can be used in the control of *B. cinerea* in strawberry, raspberry and tomato [4]. However, little is known about the non-host defense response mechanisms and defenses of tomato leaves treated with *C. rosea*.

Many defense enzymes are involved in the defense reaction against plant pathogens. These include oxidative enzymes such as polyphenol oxidase (PPO), which catalyzes the formation of lignin, and other oxidative phenols that contribute to the formation of defense barriers by reinforcing the cell structure [5]. Enzymes such as phenylalanine ammonia lyase (PAL) are involved in phytoalexin or phenolic compound biosynthesis. Such enzymes have been reported to function in defense responses against pathogens in several plant species [6]. Glutathione S-transferases (GSTs) play roles in both normal cellular metabolisms and the detoxification of a wide variety of xenobiotic compounds [7]. Such enzymes function in defense against pathogens in several plant species [6], [7]. Phytohormones are not only instrumental in regulating developmental processes in plants, but they also play important roles in the plant's responses to biotic and abiotic stresses. Nitric oxide (NO) and hydrogen peroxide (H_2_O_2_) are important signaling molecules that participate in the regulation of several physiological processes [8]. ROS, including superoxide (O_2_^−^) and H_2_O_2_, are generated following the recognition of a variety of pathogens, and they function as a threshold trigger for the hypersensitive response (HR) [9].

Our goal was to identify and utilize a preventive control mechanism to control gray mold inside tomato fruit. First, we inoculated tomato leaves with *B. cinerea* to study its prevention effect and resistance mechanism through the activities of the following molecules that function in tomato metabolism: enzymes including PAL, PPO and GST; secondary messengers including O_2_^−^, H_2_O_2_ and NO; phytohormones including indoleacetic acid (IAA), abscisic acid (ABA), gibberellins 3 (GA_3_), zeatin (ZT), jasmonic acid (JA), salicylic acid (SA), methyl jasmonate (MeJA) and ethylene (ET); and the expression of mitogen-activated protein kinase (MAPK) and WRKY genes. We used two-dimensional gel electrophoresis to evaluate protein activities during the defense process. The results of this study help elucidate the biological control and non-host resistance mechanisms of *C. rosea* as well as find the key protein involved in plant defenses. Moreover, we demonstrate the potential of *C. rosea* in controlling gray mold in tomato leaves and identify the genes that can improve tomato resistance to pathogens.

## Materials and Methods

### Cultivars tested

The homozygous tomato line 704f was used in this study; seeds were propagated in the horticultural experimental station at Northeast Agricultural University, Harbin, China.

### Microbial culture

Strain antagonist *C. rosea* was isolated from turfy soil in the suburbs of Jilin City (Northeast region of China) and deposited into the China General Microbiological Culture Collection Center (CGMCC). *C. rosea* was cultured on potato dextrose agar (PDA) plates at 22°C.

*B. cinerea* was isolated from infected tomato plants grown in the greenhouse and cultured on PDA at 25°C. The conidia were suspended in distilled water containing 0.01% Tween, 0.01% glucose and 0.01 mol·L^−1^ KH_2_PO_4_ (pH 5.0), and the concentration of spores was adjusted to 10^7^ spores·mL^−1^.

### Fungal treatment and infection

Three treatments, including *B. cinerea* treatment, *C. rosea* treatment and *B. cinerea* plus *C. rosea* treatment, were utilized in this study.

When the plants contained 5–6 leaves, the third leaf with its petiole was detached and washed with sterile distilled water and dried on filter paper. The leaves were then treated with *B. cinerea* conidia suspension, *C. rosea* conidia suspension or *B. cinerea* conidia suspension plus *C. rosea* conidia suspension. In the *B. cinerea* plus *C. rosea* treatment, the leaves were first treated with *B. cinerea* conidia suspension, and were then treated with *C. rosea* conidia suspension. For the control, the tomato leaves were treated with water. Fifteen leaves were used per treatment, with three replications.

### Determination of activities related to defense

After treatment, the tomato leaves were immediately transferred to an air-tight plastic bag to maintain a high relative humidity level and incubated at 25°C. The activity related to defense was determined by sampling the tomato leaves with each treatment administrated at an interval of 12 h to 96 h. Treated leaf samples were examined for their enzymatic activity. The effect of *C. rosea* on tomato leaves to control gray mold was examined by extraction of defense-related enzymes. Each experiment was repeated three times.

### Enzyme activity assay of PAL, PPO and GST in tomato leaves

For the enzyme assays, fresh leaf tissues were collected at different time points after treatment. All enzyme extraction procedures were conducted at 4°C. To analyze PAL activity, 0.5 g of leaves was ground in 5 mL of extraction buffer (0.1 mol/L boric acid buffer, 2.0 mmol/L mercaptoethanol, 0.5 g of polyvinypoly-pyrrolidone [PVPP], 6 mol/L HCl, pH 8.8) in an ice bath. The extracts were then homogenized and centrifuged at 10,000 rpm at 4°C for 30 min, and the supernatant was collected and used as the enzyme source. Then, 1 mL of the enzyme extract was incubated with 2 mL of 0.01 mol/L boric acid buffer solution (pH 8.8), 1 mL of 0.02 mol/L-phenylalanine and 1 mL of water, mixed and incubated in a water bath at 30°C for 60 min, followed by the addition of 0.2 mL of 6 mol/L HCl to terminate the reaction [10]. The absorbance at 290 nm was measured in an ultraviolet spectrophotometer. One unit of PAL activity equals an increase 0.01 in the UV light absorbance at 290 nm.

For PPO activity analysis, 1 g of leaves was ground in 5 mL of 100 mM sodium phosphate buffer (pH 6.4) containing 0.2 g of PVPP in an ice bath. The extracts were then homogenized and centrifuged at 12,000 rpm at 4°C for 20 min and the supernatant was collected and used as the enzyme source. Then, 0.1 mL of extract was incubated with 2 mL of phosphate buffer solution (0.05 mol/L, pH 5.5) and 0.5 mL of 0.5 M catechol solution at 24°C for 2 min [11]. The absorbance at 525 nm was measured with an ultraviolet spectrophotometer. One unit of PAL activity equals an increase of 0.01 UV light absorbance at 525 nm.

For the GST activity test, 1 g of leaves was ground in 10 mL of buffer solution (0.1 mol/L Tris-HCl (pH 7.8), 0.5 mmol/L EDTA, 0.5 mmol/L mercaptoethanol and 1% polyvinyl pyrrolidone [PVP]). The extracts were then centrifuged at 2,000 rpm at 4°C for 10 min, and the supernatant was centrifuged at 12,000 rpm at 4°C for 10 min. The supernatant was collected and used as the enzyme source. For the GST assay analysis, 1-chloro-2,4-dinitrobenzene (CDNB) was used as the substrate; 30 µL of enzyme extracts were incubated with 0.9 mL of 3.3 mmol/L glutathione (GSH), 1.97 mL 100 mmol/L potassium phosphate buffer (pH 6.5) and 100 µL of 30 mmol/L CDNB (dissolved in ethanol) [12]. The absorbance was recorded between 90 and 120 s at 340 nm. Reaction mixture without enzyme was used as a control. The GST activity was expressed as U/mg of protein.

### Determination of the second messengers: NO, H_2_O_2_ and O_2_^−^

Superoxide radical (O_2_^−^) production was assessed according to the method of Wang & Lou [13]. Briefly, 0.5 g leaves were ground in phosphate buffer (65 mM, pH 7.8) and centrifuged at 10,000 rpm for 10 min. Then, 0.5 mL supernatant was mixed with 0.5 mL phosphate buffer and 0.1 mL hydroxylamine hydrochloride (10 mM), and reacted at 25°C for 20 min. After that, 1 mL of 17 mM p-aminobenzene sulfonic acid and 7 mM alpha-aminonaphthalene was added and allowed to react for 20 minutes at 25°C. The mixture was then extracted with ether. The absorbance values of the aqueous phase were measured at 530 nm after stratification. A standard curve with NO_2_ was established to calculate the production rate of O_2_^−^ from the chemical reaction of O_2_^−^ and hydroxylamine.

The quantification of hydrogen peroxide (H_2_O_2_) in extracts from tomato leaves was performed according to Patterson [14]. Firstly, 0.5 g of leaves was ground to a homogenate using a mortar and pestle. The homogenates were centrifuged at 10,000 rpm for 10 min and 0.1 mL of concentrated hydrochloride containing 20% (V/V) TiCl_4_ and 0.2 mL of strong aqua ammonia was added to every 0.5 mL of supernatant. The mixture was centrifuged at 6,000 rpm for 10 min, and the supernatant was discarded. The titanium peroxide complex produced was washed five times with acetone. The absorbance of a titanium peroxide complex was measured at 410 nm. A standard curve of H_2_O_2_ was established according to the production rate of the O_2_^−^.

The extraction of nitrite was performed using the procedure described by Misko [15]. Briefly, 0.4 g leaves were ground to a powder using liquid nitrogen and a mortar and pestle. Then, 1 mL distilled water and 180 µL 1 M NaOH solution were added and ground to homogenates. The homogenates were transferred into a 5 mL tube, and 180 µl 1 M ZnSO_4_ solution was added and blended. The solution was incubated at 65°C for 15 min after the distilled water was added in the 5-mL solution. The solution was transferred to a 50 mL centrifuge tube, and centrifuged at 6,000 rpm for 15 min. The supernatant was transferred into a 5 mL centrifuge tube and 1 mL CCL_4_/CHCL_3_ (3∶1) solution was added to remove the proteins and pigment. The solution was mixed thoroughly by shaking and centrifuged at 6,000 g for 1 min. Finally, 2.4 mL of the supernatant was mixed with Griess A solution (l% p-aminobenzenesulfonamide dissolved in 5% phosphoric acid) and Griess B (0.1% n-(1-naphthyl)-ethylenediamine dihydrochloride) solution, and made up to 5 mL with distilled water. The absorbance of the sample solution was measured at 548 nm after 25 min incubation at dark condition. A standard curve of NO was established using different concentrations of NaNO_2_.

For these experiments, each experiment was repeated three times.

### Determination of phytohormone contents: IAA, ABA, GA3, ZT, MeJA, SA and C_2_H_4_

The determination of IAA, ABA, GA_3_ and ZT contents was performed on the same sample. Samples of leaves collected from the various treatments were cleaned and dried with a paper towel, immediately weighed and frozen in liquid nitrogen and stored at −60°C. A total of 0.5 g of fresh sample was ground in liquid nitrogen, homogenized and extracted for 12 h with 20 mL 80% cold aqueous methanol (<0°C) in the dark at 4°C. The extract was centrifuged at 5,000 rpm and 4°C for 15 min and the supernatant was collected. Then, fresh, cold methanol was poured into the residue, which was extracted three times according to Chen & Yang [16]. The total methanolic extract was dried in rotary evaporator and dissolved in 10 mL methanol. IAA, ABA, GA_3_ and ZT were analyzed by HPLC chromatography using a wavelength of UV-254 nm, a 150 mm×4.9 mm column, 0.45 µm C_18_HICHROM316A-LOK -LOK (KU), 0.6% acetic acid and a flow rate of 0.8 mL/min, a column temperature of 40°C and a sample volume of 20 µL.

#### MeJA and SA

Leaf tissues (0.5 g) from the different treatments were ground in liquid nitrogen, homogenized and then extracted for 12 h with 15 mL 80% cold aqueous methanol. After centrifugation (15 min at 1,400×g), the residue was extracted again with 100% methanol (0.5 mL) containing 10% ethyl acetate and 1% acetic acid (V/V). The combined extract was used for quantification of free SA and MeJA. MeJA and SA were separated using HPLC (high-performance liquid chromatography); chromatographic separation was carried out with a 5 µm C_18_ column (250 nm×4 nm) at room temperature.

Ethylene production was determined using gas chromatography (Hewlett-Packard, Avondale, PA, USA) as described by Hartmond [17].

For these experiments, each experiment was repeated three times.

### Semi-quantitative RT-PCR of *MAPK* and *WRKY* gene

The leaf samples were collected from different treatments. Total RNA was extracted using TRIzol Reagent (Invitrogen, USA) according to the manufacturer's instructions. Total RNA was dissolved in 20 µL of RNase free H_2_O, quantified by spectrophotometry and stored at −80°C.

In brief, 8 µL total RNA extracted from triplicate of tomato leaves was reverse-transcribed with Easyscript first-strand cDNA synthesis superMix (Beijing Transgen Biotech Co. Ltd, Beijing) according to the manufacturer's protocol and stored at −80°C respectively. Semi-quantitative PCR was performed to study the gene expression change of *MAPK* (GenBank accession number: NM_001247082.1) and *WRKY* (GenBank accession number: NM_001247468.1). The *β-actin* gene was used as the reference gene and amplified using the CTTGAAATATCCCATTGAGCA and TCAGTCAGGAGAACAGGGTG (5′–3′) primers. The primers GATGCTCATTTGCACCTGGTTGC and TCCTGATATGGCGGCAGCAAGTG, GGTTCCGTTCCGCAAACGGATAC and CTGGCAGTGCTCCTCAGATAAAC were used to amplify *WRKY* and *MAPK*, respectively.

Each PCR reaction (50 µL) contained 2 µL cDNA, 1 µL of each primer (10 µM), 5 µL 10× buffer, 3 µL MgCl_2_, 4 µL 2.5 mmol/L dNTPs, 0.5 µL Taq enzyme and 31.5 µL ddH_2_O. The RT-PCR was performed as follows: 94°C for 5 min, 35 cycles of 94°C for 30 s, 55°C for 30 s and 72°C for 1 min, followed by extension at 72°C for 10 min. Each PCR reaction was conducted three times.

### Two-dimensional gel electrophoresis

Approximately 1 g of leaves from each treatment was ground in liquid nitrogen. The crushed samples were transferred into a 50 mL centrifuge tube and mixed with three volumes of ice-cold buffer A, comprising 10 mL 10% (W/V) trichloroacetic acid (TCA), 70 µL 0.07% (V/V) β-mercaptoethanol, and 100 µL precooled acetone (under −20°C) plus ddH_2_O to a final volume of 100 mL. Protease inhibitor mixture was added at a concentration of 1% (V/V), and the mixture was incubated at −20°C overnight. After centrifugation at 40,000 rpm for 1 h at 4°C, the supernatant was mixed with three volumes of ice-cold acetone and incubated at −20°C for 1 h. The proteins were sedimented by centrifugation at 4°C, 40,000 rpm/min for 1 h and dried in a vacuum. The dried powder was transferred into a 10 mL centrifuge tube and dissolved in buffer B, which contained 7 mol/L urea (16.8 g), 2 mol/L thiourea (6.08 g), 4% (W/V) CHAPS (1.60 g), 40 mmol/L of DTT (0.248 g) and ddH2O to a final volume of 40 mL. A total of 1% (V/V) protease inhibitor mixture was added to the mixture, along with 2% Pharmalyte 3–10 ampholytes (30 µL/mg.). The mixture was incubated on ice for 1 h with stirring. The insoluble material was pelleted by centrifugation at 4°C at 40,000 rpm for 1 h.

The concentration of the proteins was determined using a 2-D Quant Kit (GE Healthcare, Shanghai) following the manufacturer's instructions.

Each sample was subjected to three replicate procedures; for each replicate, 1,000 µg of protein (resuspended in 450 µL rehydration solution, GE Healthcare) was loaded onto a 24 cm IPG Strip, pH 4 to 7 (GE Healthcare) that had been rehydrated for 15 h. The immobilized pH gradient IPG strips were then subjected to IEF at 20°C with a current of 50 µA/strip in an Ettan IPGphor isoelectric focusing apparatus (Amersham Biosciences). The voltage settings for IEF were as follows: 30 V for 8 h, 50 V for 4 h, 100 V for 1 h, 300 V for 1 h, 500 V for 1 h; 1,000 V for 1 h; 8,000 V for 12 h.

After IEF, the strips were equilibrated for 15 min in 5 mL equilibration buffer A (50 mM Tris-HCL, [pH 8.8], 6 M urea, 30% glycerol, 2% SDS, 0.01% bromophenol blue and 1% DTT). The strips were washed twice with distilled water and further equilibrated with buffer B (50 mM Tris-HCL, [pH 8.8], 6 M urea, 30% glycerol, 2% SDS, 0.002% bromophenol blue and 2.5% iodoacetamide) for 15 min prior to SDS-PAGE. The strips were then placed onto a 12.5% SDS polyacrylamide gel and covered with 0.5% agarose; the separation in the 2nd dimension was performed using Ettan Dalt SIL ELECT UNIT 230 electrophoresis apparatus. The gels were run at 2 W at 18°C for 5–6 h.

After electrophoresis, the gels were rinsed with distilled water and fixed for 30 min in 50% ethanol and 5% acetic acid solution. The gels were then enlarged in 10% acetic acid for 20 min. The gel was stained with 0.04% (w/v) Coomassie blue R-350 in 10% acetic acid for 10 min. Finally, the gels were destained with 10% acetic acid for 2–3 h.

Image acquisition was performed using a UMAX Scanner, which allowed images to be captured electronically; the analysis software Image Master 2-D TM Elit (Version 4–10) was used to analyze the images obtained from the two-dimensional gel electrophoresis.

### Mass spectrometry of proteins

The protein spots of interest were excised from the gels and placed into 500 µl Eppendorf tubes. The gel pieces were washed with 50 µl ddH_2_O and then destained with 50 µl of 50% (V/V) 50 mM ammonium bicarbonate and 50% (v/v) acetonitrile, with rotation, for 1 h. Then, 50 µl acetonitrile was added to dehydrate the gel pieces for 15 min, which were then dried in a SpeedVac (Savant instruments, Holbrook, NY, USA) until they turned white. Then, 4 µl of digestion solution (25 mM ammonium bicarbonate containing with 5 ng/µl trypsin; Roche, Indianapolis, IN, USA) was added to the dry gel pieces obtained above and rehydrated at 4°C until the gel pieces were saturated with the digestion solution. After enzymolysis for 12–14 h at 37°C, 6–8 µl of 0.5% (V/V) trifluoroacetic acid (TFA) was added and the mixtures were incubated, with rotation, for 1 h. The peptides were extracted in acetonitrile for 1 h at 37°C and then in TFA/acetonitrile for 1 h at 37°C with rotation.

After the two TFA solutions were centrifuged, 1 µL of the residue was dissolved in 1 µL of 50% acetonitrile/0.1 TFA, which contained 10 mg/mL CHCA. MS analysis was then performed following the method described by Bi [18] using a mass spectrometer (Ultraf. lexTMMALDI-T0F-MS/M), and the PMF obtained were Analyzed by NCBInr (http://www.matrixscience.co.vk).

### Real-time PCR of *atpA* and *Lexyl2* gene

The leaf samples were collected from different treatments. Total RNA was extracted using TRIzol Reagent (Invitrogen, USA) according to the manufacturer's instructions. Total RNA was dissolved in 20 µL of RNase free H_2_O, quantified by spectrophotometry and stored at −80°C. Then, 8 µL total RNA extracted from tomato leaves was reverse-transcribed with Easyscript first-strand cDNA synthesis supermix (Beijing Transgen Biotech Co. Ltd, Beijing) according to the manufacturer's protocol and stored at −80°C before use.

Bio-Rad Super SYBR Green mix was used for the reaction. Each PCR reaction for two types of samples (treatments and control) and two genes (reference and target) were conducted in triplicate. Each PCR reaction (20 µL) contained 10 µL Bio-Rad Super SYBR Green mix, 2 µL cDNA (25 ng), 0.6 µL each primer (10 µM) and 6.8 µL ddH_2_O. The PCR reactions were dispensed into ABI optical reaction tubes (Applied Biosystems, Foster City, CA, USA). The reaction tubes were centrifuged at 2,500 rpm for 10 s to settle the reaction mixtures to the bottom of the wells. PCR was carried out with an iCycler real-time quantity PCR system (Bio-Rad). The RT-PCR was performed as follows: 94°C for 3 min, 1 cycle, 95°C for 45 s, 52°C for 45 s, 72°C for 60 s, 35 cycles and 72°C for 10 min. After each run, a dissociation curve was designed to confirm specificity of the product and to avoid production of primer-dimers. All statistical analyses were performed with the 2^−ΔΔCt^ methods. The sequences used for *β-actin* amplification were CCACCTTAATCTTCATGCTGCT and ACATTGTGCTCAGTGGTGGTACT. The sequences used for β-xylosidase (*Lexyl2*) gene (GenBank accession number: AB041812.1) amplification were GTGGTGTTTGTATTGGGTGT and GTGGTGCTGCGTTGGCTGA. The sequences used for ATP synthase CF1 α subunit (*atpA*) gene (GenBank accession number: AC_000188.1) amplification (reverse primer and forward primer) were GAGTGAGGCTTATTTGGGTC and AGGCTCATATACGGAACGG. The primer sequences used for *β-actin* amplification were those published by Wang [19]. The primer sequences used for *atpA* and *Lexyl2* were found on the NCBI site.
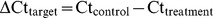
(1)

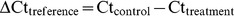
(2)

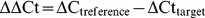
(3)

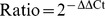
(4)


In which the target genes are *atpA* and *Lexyl2*, and the reference gene is β–*actin*.

### Statistical analysis

Statistical analysis was performed using statistical program from social sciences (SPSS) Version 17.0. Data were analyzed using one-way ANOVA. Separations were performed by Duncan's multiple range tests. Differences at P<0.05 were considered to be significant. The means and sample variance were equal in all experiments.

## Results

### The effect of *C. rosea* treatment on PAL, PPO and GST activities in tomato leaves

**Figure 1:**
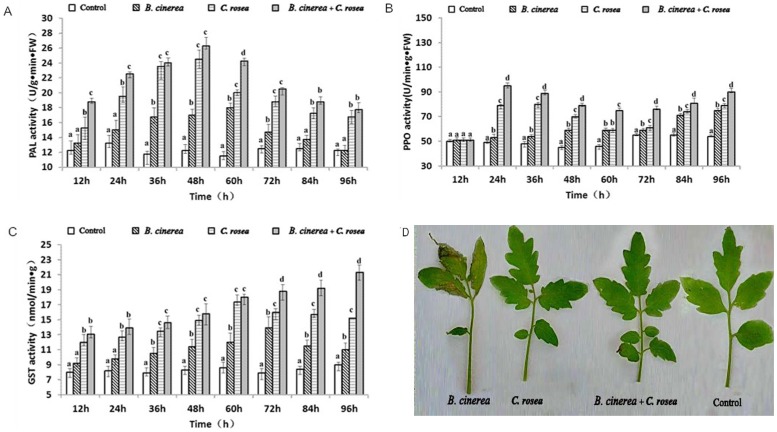
Changes in the activities of resistance-related enzymes in tomato leaves subjected to different treatments. Open bars indicates water control (Control), left-diagonal hatched bars indicates plants treated with *B. cinerea* (10^7^ cfu spores/mL), horizontal bars indicates plants treated with C. rosea (107 cfu spores/mL) alone, right-diagonal hatched bars indicates plants inoculated with *B. cinerea* (10^7^ cfu spores/mL) before the application of *C. rosea* (10^7^ cfu spores/mL). Each experiment was repeated three times. Data are presented as mean ± S.D. Means marked with different letters (a, b, c) are significantly different at P<0.05. A: Changes in PAL activity in tomato leaves subjected to different treatments. B: Changes in PPO activity in tomato leaves subjected to different treatments. C: Changes in GST activity in tomato leaves subjected to different treatments. D: Prevention effects of *C. rosea* to *B. cinerea* at 60 h after treatment.

PAL activities increased at the different degrees in all three treatments compared to the control. In *C. rosea* treatment and *B. cinerea* plus *C. rosea* treatment, the PAL activity shifted and increased gradually, reaching its peak at 48 h, with the maximum values of 100% and 114.3% higher than that of the control, respectively. The PAL activity in leaves inoculated with *B. cinerea* alone increased before reaching its peak at 60 h, with a value of 56.5% (Fig. 1.A). The maximum value of PAL activity in leaves treated with *B. cinerea* plus *C. rosea* was 7.14% higher than that of leaves treated with *C. rosea* alone (Fig. 1A). At all the same time points, PAL activities after *B. cinerea* plus *C. rosea* treatment were highest among all three treatments, followed by those after *C. rosea* treatment. These results showed that treatment with *B. cinerea* plus *C. rosea* stimulated the activity of PAL enzymes in leaves most significantly. These results also indicated that the PPO activity for the three treatments did not change within 12 h. At 12 h (Fig. 1B), the PPO activity in leaves treated with *C. rosea* alone gradually increased and reached its peak at 36 h, with a maximum value of 35.6%. The PPO activity in *B. cinerea* treatment also progressed but at a steady rate within 24 h to 36 h. The time points when PPO activity reached the peak in *B. cinerea* treatment and *B. cinerea* plus *C. rosea* treatment were 24 h and 84 h earlier than in the *B. cinerea* treatment. The maximum value of PPO in leaves inoculated with *B. cinerea* and treated with *C. rosea* was 18.75% higher than that of leaves treated with *C. rosea* alone (Fig. 1B). After inoculating *B. cinerea*, the GST activity gradually increased, reaching its peak at 72 h but gradually declining thereafter. The GST activity in leaves inoculated with *C. rosea* alone increased after reaching its peak at 60 h, with a value of 120.25% higher than that of the control, followed by a gradual decline. The GST activity in inoculated leaves treated with *C. rosea* showed a gradual increase within 96 h, with a maximum value of 136.67%, and was still higher than that of the other two treatments (Fig. 1C.).

### Changes in secondary messengers

**Figure 2:**
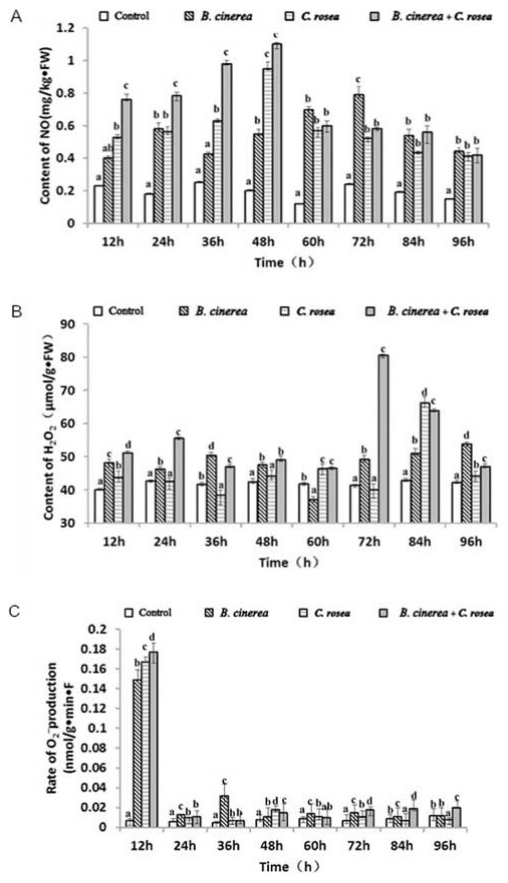
Changes in second messenger levels in tomato leaves subjected to different treatments. Open bars indicates water control, left-diagonal hatched bars indicates plants treated with *B. cinerea* (10^7^ cfu spores/mL), horizontal bars indicates plants treated with *C. rosea* (10^7^ cfu spores/mL) alone, right-diagonal hatched bars indicates plants inoculated with *B. cinerea* (10^7^ cfu spores/mL) before the application of *C. rosea* (10^7^ cfu spores/mL). Each experiment was repeated three times. Data are presented as mean ± S.D. Means marked with different letters (a, b, c) are significantly different at P<0.05. A: Changes in NO levels in tomato leaves subjected to different treatments. B: Changes in H_2_O_2_ levels in tomato leaves subjected to different treatments. C: Changes of O_2_^−^ levels in tomato leaves subjected to different treatments.

The control leaves did not contain a large amount of H_2_O_2_, which was maintained at a constant level, but tomato leaves treated with *B. cinerea* showed an increase in H_2_O_2_ levels at 12 h. However, the increase was not exponential because within 48 h, we observed a relatively stable state, which was followed by a decrease; the maximum value was 53.8 µmol/g FW at 96 h. Leaves treated with *C. rosea* showed no significant change in H_2_O_2_ levels, but 72 h later, a rapid increase was observed, with a maximum value of 66.2 µmol/g FW at 84 h. Leaves treated with *C. rosea* and inoculated with *B. cinerea* showed an increase in H_2_O_2_ levels at 24 h, followed by a steady state and then a rapid increase at 60 h. A sharp decline was noted within 72 h (Fig. 2A). We found that *B. cinerea* plus *C. rosea* treatment produced higher levels of H_2_O_2_ than the other two treatments.

The control leaves did not contain a large amount of O_2_^−^, but a significant increase in O_2_^−^ levels was observed, with *B. cinerea* plus *C. rosea* treatment producing the highest O_2_^−^ level, followed by *B. cinerea* plus *C. rosea* treatment. At 12 h, the O_2_^−^ levels increased for all treatments. At 36 h, the same level was observed for all treatments and for the control (Fig. 2B).

Control tomato leaves treated exhibited a stable, low level of NO. The three treatments each produced a significant maximum value of NO content. Tomato leaves treated with *B. cinerea* exhibited an increase in NO content beginning at 12 h, which declined between 24 and 36 h, followed by an exponential increase that reached a maximum at 72 h. Tomato leaves treated with *C. rosea* showed an increase in NO concentration, which reached a maximum value between 12 and 48 h, followed by a decline. Tomato leaves treated with *C. rosea* and inoculated with *B. cinerea* exhibited NO at 12 h and reach a maximum value at 48 h (Fig. 2C). The results showed that *B. cinerea* plus *C. rosea* treatment produced the highest level of NO compared to the other two treatments.

### Changes in phytohormone content

**Figure 3:**
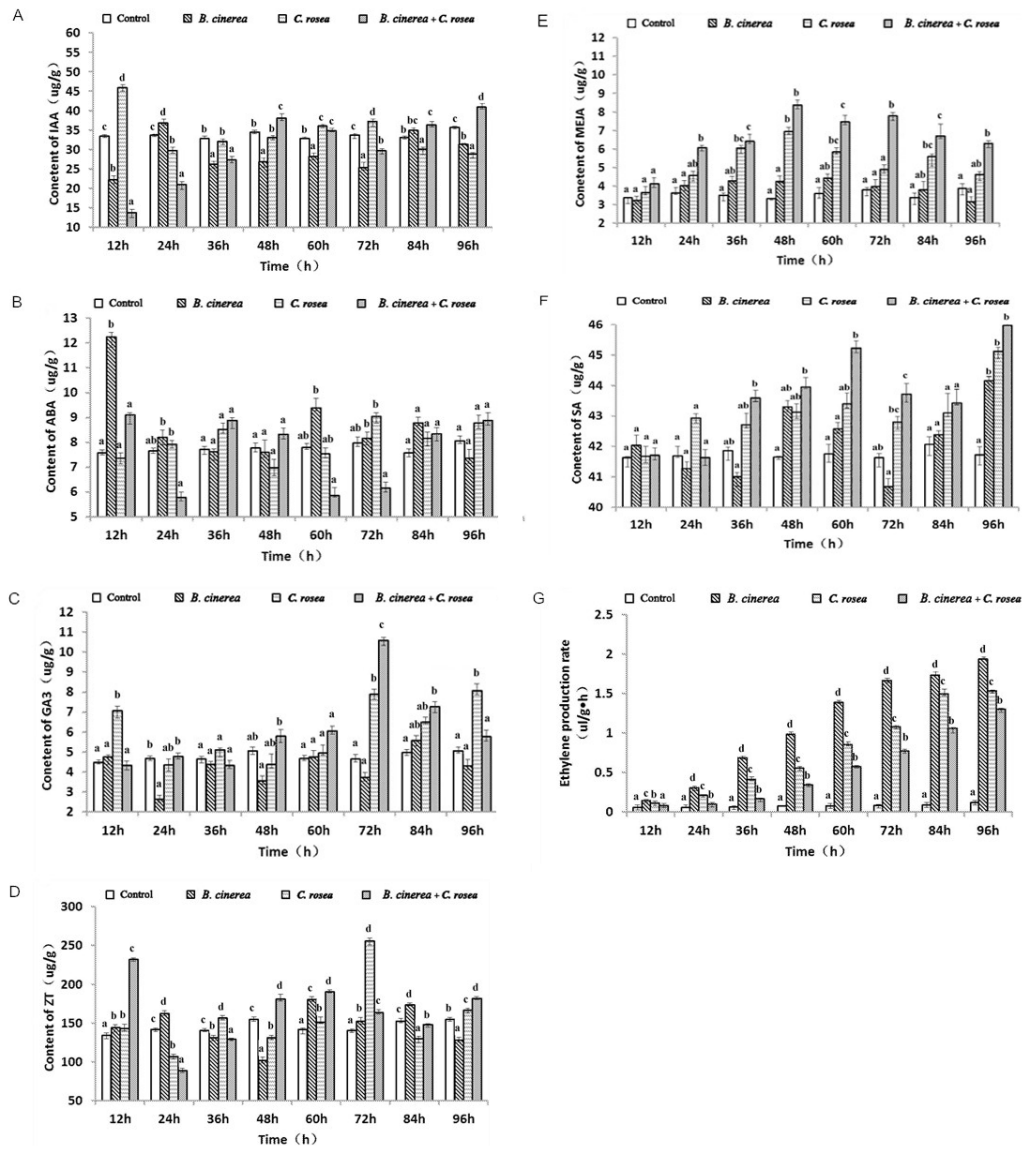
Changes in phytohormone levels in tomato leaves subjected to different treatments. Open bars indicates water control, left-diagonal hatched bars indicates plants treated with *B. cinerea* (10^7^ cfu spores/mL), horizontal bars indicates plants treated with *C. rosea* (10^7^ cfu spores/mL) alone, right-diagonal hatched bars indicates plants inoculated with *B. cinerea* (10^7^ cfu spores/mL) before the application of *C. rosea* (10^7^ cfu spores/mL). Each experiment was repeated three times. Data are presented as mean ± S.D. Means marked with different letters (a, b, c) are significantly different at P<0.05. A: Changes in IAA levels in tomato leaves subjected to different treatments. B: Changes in ABA levels in tomato leaves subjected to different treatments. C: Changes in GA3 levels in tomato leaves subjected to different treatments. D: Changes in ZT levels in tomato leaves subjected to different treatments. E: Changes in MeJA levels in tomato leaves subjected to different treatments. F: Changes in SA levels in tomato leaves subjected to different treatments. G: Changes in C_2_H_4_ levels in tomato leaves subjected to different treatments.

The IAA content in the controls was stable during the 96 h treatment period, with no large fluctuations observed; after the leaves were inoculated with *B. cinerea*, the IAA content decreased at 12 h, increased again, and then declined. We observed a second peak, but the increase was quite small, and in most cases it was less than the control level; after the leaves were inoculated with *C. rosea*, the IAA content significantly increased at 12 h, and although we observed a decrease at some point, the level increased immediately at 24 h. For leaves treated with *C. rosea* and inoculated with *B. cinerea*, the IAA levels decreased obviously at 12 h, reaching levels even lower than the levels observed in *B. cinerea* treatment, but at 12 h, the level began to rise, and at 36 h, the levels were higher than those observed in *B. cinerea* treatment, with all fluctuations close to the control (Fig. 3A).

Tomato plants in the control group showed no significant change in ABA content, which was maintained at a steady level. Tomato leaves treated with *B. cinerea* showed the highest ABA content, and a rapid increase in the ABA content was observed within 12 h after inoculation, followed by a large decline. Leaves treated with *C. rosea* had an ABA content close to that of the control, and some levels were even below those of the control. In leaves treated with *C. rosea* and inoculated with *B. cinerea*, a high content of ABA relative to that of the control was observed at 12 h and was much lower than that of *B. cinerea* treatment, and the level also exhibited a lower decline than that of the control (Fig. 3B).

As shown in the figure, control tomato leaves exhibited a stable content of GA_3_, and tomato leaves treated with *B. cinerea* had a GA_3_ content close to or even lower than that of the control. In tomato leaves treated with *C. rosea*, the content of GA_3_ began to change at 12 h, reaching a maximum value at 96 h. The GA_3_ levels in tomato leaves treated with *C. rosea* and inoculated with *B. cinerea* did not change during the 36 h period after inoculation, but at 48 h, an exponential increase was observed, with a maximum value observed at 72 h, which was followed by a decline that was less than that of the control; the maximum value of GA_3_ content was higher than that of the other two treatments (Fig. 3C).

Tomato leaves treated with *B. cinerea* exhibited a highly variable change in the content of ZT, with three peaks observed at three different periods and the highest level observed at 60 h. Leaves treated with *C. rosea* showed no change in ZT content at 60 h, but a rapid increase in ZT content was subsequently observed, with the maximum ZT level reached at 72 h, followed by a lower decline than that of the control. Leaves treated with *C. rosea* and inoculated with *B. cinerea* exhibited a higher content of ZT followed by a lower decline compared to that of the control, but the level subsequently tended to increase. The ZT content was higher in *B. cinerea* treatment and the control, but the levels in *C. rosea* treatment and *B. cinerea* plus *C. rosea* treatment were similar to those of the control (Fig. 3D).

The MeJA content of the control leaves was relatively stable. The content of MeJA in tomato leaves treated with *B. cinerea* showed little change and was almost identical to that of the control. Treatments *C. rosea* and *B. cinerea* plus *C. rosea* exhibited almost the same changes in MeJA content, and the contents in both treatment groups reached a maximum value at 48 h, but the maximum value and changes of these three treatments were different. However, among the different treatments, *B. cinerea* plus *C. rosea* treatment produced the greatest value (Fig. 3E).

The SA content in the control leaves was fairly stable, and the levels among all three treatment groups were similar at 12 h. In tomato leaves treated with *B. cinerea*, we observed a change in SA content at 36 h, with an exponential increase observed, followed by a lower decrease than was observed in the control, with a maximum value of 44.16 µmg/g observed at 96 h. Leaves treated with *C. rosea* showed a change in the content of SA at 12 h, reaching a maximum value of 45.12 µmg/g at 96 h, but between 60 and 72 h, the level fell. In leaves inoculated with *B. cinerea* and treated with *C. rosea*, the content of SA was fairly constant for almost 24 h and was almost identical to that of the control. A significant change in SA content was observed at 60 h, with a value of 45.23 µg/g, followed by a decline, subsequently reaching a maximum value of 45.98 µg/g at 96 h. All three treatments produced a maximum value at 96 h, with the highest SA level produced by *B. cinerea* plus *C. rosea* treatment (Fig. 3F).

The ethylene content of the control leaves was stable, while all three treatments produced exponential increases in the content of ethylene. All three treatments exhibited their maximum values at 96 h, and *B. cinerea* treatment produced the greatest value. These results indicate that infection of tomato leaves by *B. cinerea* induces the biosynthesis of ethylene and increases the content of ethylene (Fig. 3G).

### Expression of *MAPK* gene

**Figure 4:**
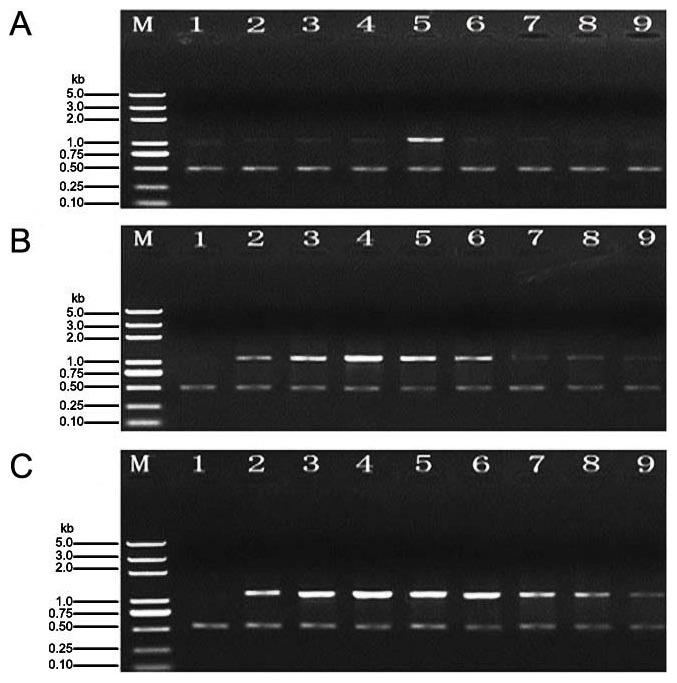
*MAPK* gene expression in tomato leaves subjected to different treatments. Note: The time points (1–9) after treatment were 0 h, 24 h, 36h, 48 h, 56 h, 60 h, 72 h and 84 h, respectively. The experiments were repeated three times, 0 h stand for the control. A: The relative expression level of the *MAPK* gene in *B. cinerea* treatment. B: The relative expression level of the *MAPK* gene in *C. rosea* treatment. C: The relative expression level of the *MAPK* gene in *B. cinerea* plus *C. rosea* treatment.

We observed the sizes of MAPK gene amplification products at different sampling times, including 0 h, 12 h, 24 h, 36 h, 48 h, 56 h, 60 h, 72 h and 84 h, The 0 h time point represents the size of the *MAPK* gene amplification products in tomato leaves treated with distilled water (control; Fig. 4).

The amplification products in the *B. cinerea* treatment were first clearly observed at 24 h. At 48 h, we observed the strongest expression of the MAPK gene, after which the expression became progressively weaker until it reach a minimum level at 84 h (Fig. 4A). The amplification product bands in *C. rosea* treatment were first observed at 12 h, with the highest expression level observed at 36 h, after which the expression gradually decreased, reaching a minimum at 84 h (Fig. 4B).The highest expression level was observed at 36 h, and the highest expression level was maintained between 36 and 56 h for *B. cinerea* plus *C. rosea* treatment (Fig. 4C). In addition, the duration of *MAPK* gene expression in *B. cinerea* plus *C. rosea* treatment was highest in all three treatments. Overall, the duration of raised *MAPK* expression in *C. rosea* treatment was longer than found in *B. cinerea* treatment.

### Expression of *WRKY* gene

**Figure 5:**
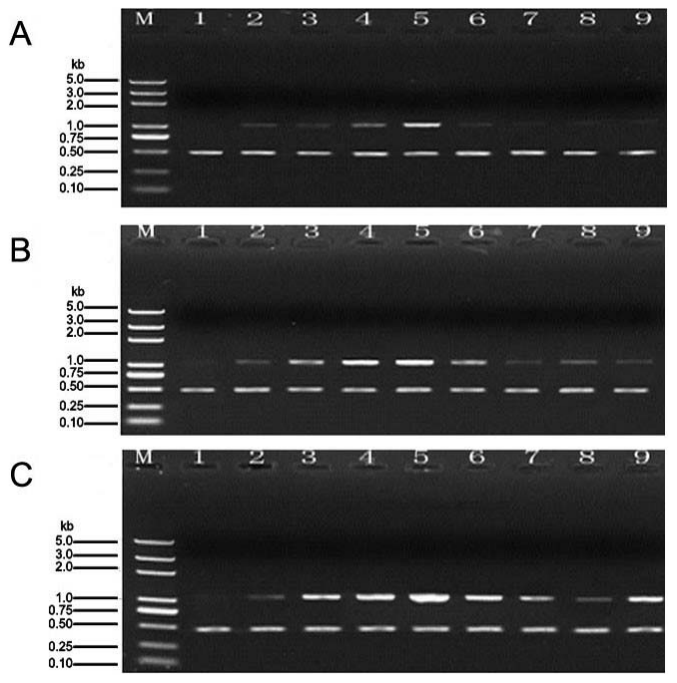
WRKY gene expression in tomato leaves subjected to different treatments. Note: The time points (1–9) after treatment were 0 h, 24 h, 36 h, 48 h, 56 h, 60 h, 72 h and 84 h, respectively. The experiments were repeated three times, 0 h stand for the control. A: The relative expression level of the *WRKY* gene in *B. cinerea* treatment. B: The relative expression level of the *WRKY* gene in *C. rosea* treatment. C: The relative expression level of the *WRKY* gene in *B. cinerea* plus *C. rosea* treatment.

We observed the expression levels of the WRKY gene amplification products at different sampling times, including 0 h, 12 h, 24 h, 36 h, 48 h, 56 h, 60 h, 72 and 84 h. The 0 h time point represents the expression levels of the WRKY gene amplification product in tomato leaves treated with distilled water (control; Fig. 5). The expression of the *WRKY* gene began to increase at 12 h and reached a peack at 48 h. After 48 h, the expression became progressively weaker; reaching the minimum levels at 84 h, but in *B. cinerea* plus *C. rosea* treatment, the expression level began to decrease at 72 h and started to increase at 84 h (Fig. 5C). *MAPK* gene expression levels in *B. cinerea* plus *C. rosea* treatment were highest of all the three treatments at all time points. Furthermore, the expression of *MAPK* in *C. rosea* treatment was higher than in *B. cinerea* treatment at all time points.

### Differentially expressed protein spots

**Figure 6:**
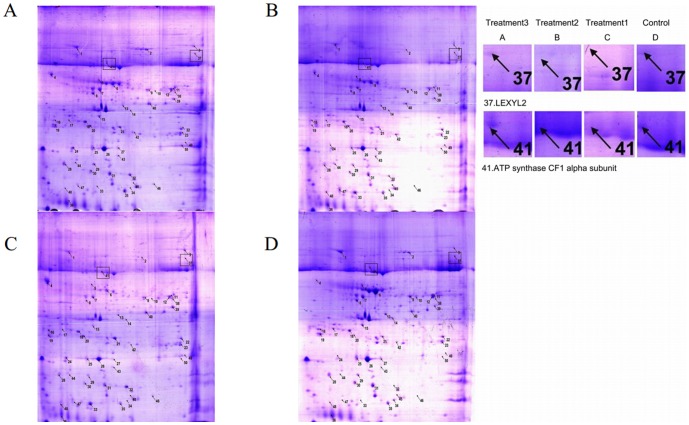
Two-dimensional gel electrophoresis analysis of proteins in leaves subjected to different treatments. A: Plants treated with *B. cinerea* (10^7^ cfu spores/mL) alone. B: Plants treated with *C. rosea* (10^7^ cfu spores/mL) alone. C: Plants inoculated with *B. cinerea* (10^7^ cfu spores/mL) before the application of *C. rosea* (10^7^ cfu spores/mL). D: Water control.

In the present study, we extracted proteins from leaf samples 72 h after treatment, as well as control leaves according to the above-described results. The proteins were investigated using mass spectrometry, as well as a UMAX Scanner, which enabled us to obtain digital images. The digital image analysis revealed the presence of 50 protein spots (Fig. 6). The protein names that represent different points are listed in Table 1.

**Table 1:** Identification of the main differentially regulated protein spots among three treatments compared to the control.

Spot No.	Protein Name	Accession No.	Species	Score	Matched Peptides	Coverage
ATP binding/unfolded protein binding						
1	Chaperone DnaK	ABE79560	*Medicago truncatula*	270	12	13%
Identical protein binding/serine-type endopeptidase activity						
3	subtilisin-like protease	CAA71234	*Solanum lycopersicum*	741	41	56%
Oxidoreductase/peroxidase						
4	Suberization-associated anionic peroxidase 1	P15003	*Solanum lycopersicum*	319	6	14%
ATP binding						
5	Ribulose bisphosphate carboxylase/oxygenase activase, chloroplastic	O49074	*Solanum pennellii*	775	16	37%
6	Ribulose bisphosphate carboxylase/oxygenase activase, chloroplastic	O49074	*Solanum pennellii*	2098	71	58%
7	Ribulose bisphosphate carboxylase/oxygenase activase, chloroplastic	O49074	*Solanum pennellii*	945	24	43%
8	Ribulose bisphosphate carboxylase/oxygenase activase, chloroplastic	O49074	*Solanum pennellii*	487	9	19%
9	Ribulose bisphosphate carboxylase/oxygenase activase, chloroplastic	O49074	*Solanum pennellii*	688	21	34%
10	Ribulose bisphosphate carboxylase/oxygenase activase, chloroplast precursor	O49074	*Solanum pennellii*	198	6	9%
44	Ribulose bisphosphate carboxylase/oxygenase activase, chloroplastic	O49074	*Solanum pennellii*	628	14	23%
Catalytic activity/coenzyme binding						
11	mRNA binding protein precursor	AAD21574	*Lycopersicon esculentum*	524	12	31%
L-malate dehydrogenase activity/binding						
12	mitochondrial malate dehydrogenase	AAU29198	*Lycopersicon esculentum*	1253	27	70%
13	mitochondrial malate dehydrogenase	AAU29198	*Lycopersicon esculentum*	236	6	17%
14	mitochondrial malate dehydrogenase	AAU29198	*Lycopersicon esculentum*	120	2	8%
Phosphatase activity						
15	Unknown/Putative uncharacterized protein	ABK95024	*Populus trichocarpa*	191	5	10%
Nucleic acid binding/nucleotide binding						
16	single-stranded DNA binding protein precursor	AAL39067	*Solanum tuberosum*	365	9	25%
RNA binding/nucleotide binding						
17	30 kDa ribonucleoprotein, chloroplastic	P49313	*Nicotiana plumbaginifolia*	91	1	4%
Transferase activity						
18	glutathione S-transferase, class-phi	AAB65163	*Solanum commersonii*	221	14	18%
23	glutathione S-transferase, class-phi	AAB65163	*Solanum commersonii*	221	14	18%
Unreviewed						
19	nascent polypeptide associated complex alpha	ACB32231	*Solanum chacoense*	281	7	44%
Carbonate dehydratase activity						
20	carbonic anhydrase	CAH60891	*Solanum lycopersicum*	198	5	12%
Threonine-type endopeptidase activity						
21	unknown	ABA81880	*Solanum tuberosum*	448	13	49%
35	Proteasome subunit beta type-1	O82531	*Petunia x hybrida*	641	20	44%
42	Proteasome subunit beta type-1	O82531	*Petunia x hybrida*	641	20	44%
Triose-phosphate isomerase activity						
22	triose phosphate isomerase cytosolic isoform-like	ABB02628	*Solanum tuberosum*	104	2	13%
Hydrogen ion transporting ATP synthase activity,rotational mechanism						
24	ATP synthase delta chain, chloroplastic	P32980	*Nicotiana tabacum*	133	4	14%
Calcium ion binding						
25	Oxygen-evolving enhancer protein 2, chloroplastic	P29795	*Solanum lycopersicum*	569	22	46%
26	Oxygen-evolving enhancer protein 2, chloroplastic	P29795	*Solanum lycopersicum*	690	35	46%
27	Oxygen-evolving enhancer protein 2, chloroplastic	P29795	*Solanum lycopersicum*	499	16	44%
30	Oxygen-evolving enhancer protein 2, chloroplastic	P93566	*Solanum tuberosum*	171	3	18%
43	Oxygen-evolving enhancer protein 2, chloroplastic	P29795	*Solanum lycopersicum*	677	16	44%
Structural constituent of ribosome						
28	ribosomal protein L12-1a	CAA44226	*Nicotiana tabacum*	410	7	38%
Hypothetical protein						
29	Hypothetical protein	AAG12570	*Arabidopsis thaliana*	237	5	15%
Defense response/response to biotic stimulus						
31	TSI-1 protein	CAA75803	*Solanum lycopersicum*	229	8	52%
32	TSI-1 protein	CAA75803	*Solanum lycopersicum*	155	6	25%
Magnesium ion binding/monooxygenase activity/ribulose-bisphosphate carboxylase activity						
33	ribulose-1,5-bisphosphate carboxylase/oxygenase large subunit	YP_514860	*Solanum lycopersicum*	260	6	10%
Nucleic acid binding/nucleotide binding						
34	SGRP-1	CAA73034	*Solanum commersonii*	138	2	16%
Monooxygenase activity/ribulose-bisphosphate carboxylase activity						
36	Ribulose bisphosphate carboxylase small chain 3A/3C, chloroplastic	P07180	*Solanum lycopersicum*	428	16	62%
Hydrolase activity,hydrolyzing O-glycosyl compounds						
37	LEXYL2	BAC98299	*Solanum lycopersicum*	418	9	18%
NAD or NADH binding/glyceraldehyde-3-phosphate dehydrogenase activity						
38	glyceraldehyde-3-phosphate dehydrogenase	CBE70550	*Manihot michaelis*	113	2	27%
Fructose-bisphosphate aldolase activity						
39	fructose-bisphosphate aldolase, putative	XP_002531508	*Ricinus communis*	710	22	27%
FAD binding/NADP or NADPH binding/ferredoxin-NADP+ reductase activity/poly(U) RNA binding						
40	Contains similarity to ferredoxin-NADP+ reductase from Arabidopsis thaliana gb|AJ243705 and contains an oxidoreductase FAD/NAD-binding PF|00175 domain.	AAF79911	*Arabidopsis thaliana*	159	5	9%
ATP binding/hydrogen ion transporting ATP synthase activity, rotational mechanism/proton-transporting ATPase activity, rotational mechanism						
41	ATP synthase CF1 alpha subunit	NP_051044	*Arabidopsis thaliana*	312	12	18%
45	ATP synthase CF1 epsilon chain	ABB90047	*Solanum tuberosum*	582	19	56%
Pathogenesis-related protein						
46	Pathogenesis-related protein STH-2	P17642	*Solanum tuberosum*	271		
magnesium ion binding/monooxygenase activity/ribulose-bisphosphate carboxylase activity						
47	ribulosebisphosphate carboxylase	AAB01597	*Solanum tuberosum*	92	3	7%
48	Ribulose bisphosphate carboxylase large chain	P28427	*Jasminum simplicifolium subsp. suavissimum*	84	2	10%
response to stress						
49	Osmotin-like protein OSML15	P50703	*Solanum commersonii*	375	5	40%
Antimicrobial/Fungicide/Pathogenesis-related protein						
50	pathogenesis-related protein PR P23	CAA50059	*Solanum lycopersicum*	276	4	36%

We noticed several differences between the protein profiles of the three treatments and the control leaves. Specifically, spots 3, 4, 20, 24, 32, 37, 39, 41, 46 and 47 were expressed in *B. cinerea* plus *C. rosea* treatment, but these spots were absent in the control. In addition, spots 4, 32, 33, 42, 44 and 47 were expressed in *C. rosea* treatment, but they were absent in the control. Moreover, spots 4, 32, 33, 44, 47, 48, 49 and 50 were only expressed in *B. cinerea* treatment but were not expressed in the control. Compared to the control, the three treatments shared some common spots, including 4, 32, 39, 44 and 47, which were specifically expressed in the treatment groups. In addition to the differences in the expression of these spots between the three treatment groups and the control, there was also a difference in the levels of expression. In *B. cinerea* plus *C. rosea* treatment, spots 2, 8, 18, 21, 28, 35, 40, 41, 42, 43 and 50 exhibited an increase in expression. During *C. rosea* treatment (compared to the control), only spots 11, 20 and 21 exhibited an increase in expression. During *B. cinerea* treatment (compared to the control), only spots 3, 18, 20, 21, 24 and 39 exhibited an increase in expression.

Spots 2, 8, 19, 37, 41, 46 and 50 were expressed in the group that was subjected to *B. cinerea* plus *C. rosea* treatment, but they were not expressed in *C. rosea* treatment. Also, spots 2, 8, 37 and 41 were expressed in *B. cinerea* plus *C. rosea* treatment but were not expressed in *B. cinerea* treatment, while spot 33 was expressed in all treatments and spots 37 and 41 (Fig. 6) were expressed only in *B. cinerea* plus *C. rosea* treatment. *B. cinerea* plus *C. rosea* treatment contained the following spots: 1, 2, 3, 6, 7, 8, 9, 12, 11, 13, 15, 16, 18, 19, 20, 22, 23, 24, 25, 26, 27, 28, 29, 30, 31, 32, 35, 36, 38, 42, 43, 46, 47 and 50. Their expression levels were higher than those of the other two treatment groups.

While all three treatments induced the expression of common proteins, they all induced the expression of specific proteins as well. Therefore, we speculate that the mechanisms of action of the three treatments are different, but share some of the same disease resistant pathways.

### Expression of *atpA* and *Lexyl* gene

**Figure 7:**
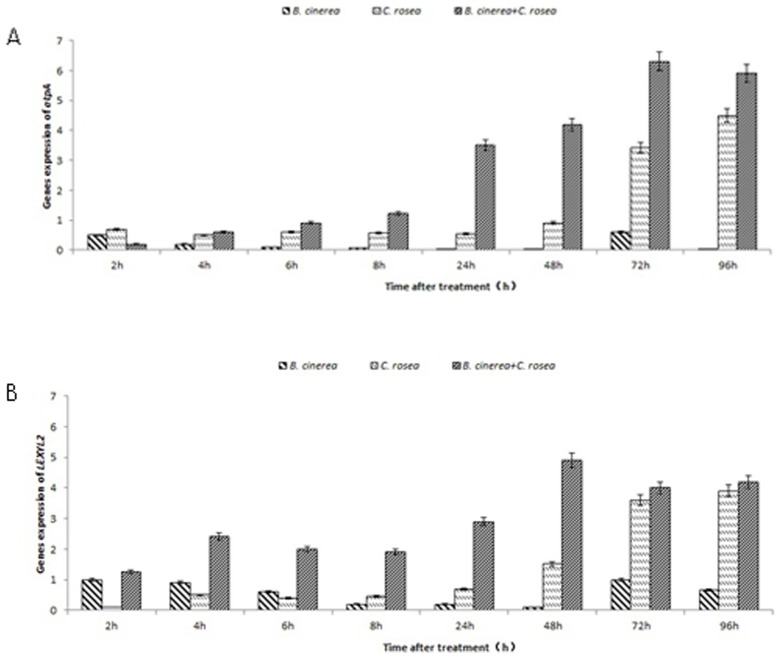
Expression of *ATP* and *LEXYL2* genes in tomato leaves subjected to different treatments. Left-diagonal hatched bars indicates plants treated with *B. cinerea* (10^7^ cfu spores/mL), horizontal bars indicates plants treated with *C. rosea* (10^7^ cfu spores/mL) alone, right-diagonal hatched bars indicates plants inoculated with *B. cinerea* (10^7^ cfu spores/mL) before the application of *C. rosea* (10^7^ cfu spores/mL). Each experiment was repeated three times. Data are presented as mean ± S.D. Means marked with different letters (a, b, c) are significantly different at P<0.05. A: Expression of *ATP* genes in tomato leaves. B: Expression of *LEXYL2* genes in tomato leaves.

As spots 37 (β-xylosidase, gene abbreviation “*Lexyl*”) and 41 (ATP synthase CF1 alpha subunit, gene abbreviation “*atpA*”) were expressed only in tomato leaves inoculated with *B. cinerea* and treated with *C. rosea*, the expression levels of these two proteins were the focus of this study. Tomato leaves treated with *B. cinerea* showed an increase in *Lexyl* expression at 2 h, followed by a decrease, with a maximum value at 72 h, while this treatment did not produce a significant change in *atpA* gene expression. Leaves treated with *C. rosea* showed no significant change in *Lexyl2* expression up to 24 h, but at 48 h, a rapid increase in gene expression was observed, with a maximum value of 3.9 observed at 96 h. The expression of *atpA* gene showed an unstable change, with a maximum value of 4.5 observed at 96 h. Leaves treated with *C. rosea* and inoculated with *B. cinerea* showed an increase in *Lexyl2* levels at 2 h, followed by a steady level and then a rapid increase, reaching a maximum value of 4.9 at 72 h. The level of *atpA* expression increased exponentially, with a maximum value of 6.3 observed at 72 h. We found that *B. cinerea* plus *C. rosea* treatment induced a higher level of *atpA* and *Lexyl2* expression than the other two treatments (Fig. 7).

## Discussion

### Change of defense enzymes in tomato leaves under treatment of *C. rosea*

The success of *C. rosea* as a biocontrol agent is believed to involve many factors and diverse modes of action [20]. Our results showed that tomato leaves treatment with *C. rosea* significantly increased the activities of the enzymes PAL, PPO and GST (Fig. 1A, B, C) and effectively inhibited gray mold formation in tomato (Fig. 1D). These results are consistent with previous findings that *C. rosea* improves resistance to *Fusarium culmorum* in wheat plants [21].

PAL is a key enzyme of the phenylpropanoid pathway that takes part in the synthesis of the phenolic compounds phytoalexin and lignin [22]. In this study, we found that the infected leaves triggered an increase in PAL activity. These results match those obtained by Ciepiela [23], who demonstrated that feeding by the grain aphid *Sitobion avenae* (F.) on aphid-resistant wheat cultivars causes an increase in PAL levels. PPO participates in the oxidation of many types of phenolic compounds, leading to the production of quinones, which are extremely toxic to several pathogens [24]. The peak levels of PPO (48.5%) after *B. cinerea* plus *C. rosea* treatment were higher than those after *C. rosea* treatment (35.6%), and we hypothesize that *C. rosea* stimulates the production of quinones in the presence of *B. cinerea* infections, which are highly toxic to pathogens.

Glutathione S-transferases (GSTs) play a role both in normal cellular metabolism and the detoxification of a wide variety of xenobiotic compounds. GST has been implicated in numerous stress responses, including those arising from pathogen attack and oxidative stress [7]. In crops such as wheat and potato, as well as *Arabidopsis thaliana*, the expression of GSTs can be induced after the plants are attacked by pathogens, indicating that GSTs play an important role in plant defense [25]–[27]. Hence, in this study, we found that the inoculation of tomato leaves by the pathogen alone caused an increase in the level of GST activity, which was also observed in tomato leaves treated with the agent antagonist alone, producing a high level of GST activity, thereby strengthening the resistance to pathogen invasion; the level of GST activity in leaves inoculated with the pathogen and treated with the agent antagonist was higher than that of the two other treatments. In concoction, treating tomato leaves with *C. rosea* increased the activities of PAL, PPO and GST; these substances are associated with the process of local disease defense.

### Change of secondary messengers in tomato leaves under *C. rosea* treatment

NO and H_2_O_2_ have been shown to be important signaling molecules that participate in the regulation of several physiological processes. In particular, these molecules play a significant role in plant resistance to pathogens by contributing to the induction of defense genes [8].

H_2_O_2_ can act as a local signal for hypersensitive cell death as well as a diffusible signal for the induction of defensive genes in adjacent cells [28]. In this study, we observed a change in H_2_O_2_ content in leaves treated with *B. cinerea* at 12 h after inoculation, which may have been caused by the outbreak of reactive oxygen species in infected tissues. H_2_O_2_ induces cell wall lignification, which improves plant resistance to pathogens [29]. Treatments *C. rosea* and *B. cinerea* plus *C. rosea* produced the greatest increase in H_2_O_2_ content compared to *B. cinerea* treatment, but all three treatments induced low H_2_O_2_ levels at 96 h, although these levels differed. This result may have been due to the excessive amount of ROS present in the plants, which have a toxic effect on plants, leading to tissue damage.

NO and reactive oxygen species play an important role in the activation of the mechanisms of disease resistance in animals and plants. However, these compounds are potentially toxic when the antioxidant system is overwhelmed and reactive oxygen intermediates (ROI) accumulate. Infection of tobacco with *tobacco mosaic* virus results in enhanced NO synthase (NOS) activity, and furthermore, administration of NO donors to tobacco plants or tobacco suspension cells triggers the expression of defense-related genes [30].

Several studies have demonstrated the effects of NO and peroxide (H_2_O_2_) on the induction of the hypersensitive response (HR) in soybean cells. These studies showed that the induction of a toxic reaction in cell depends on the effect of the synergy of these two signaling molecules. When the concentration of these molecules reaches a state of equilibrium, the HR is reduced, while if one of the signaling molecules is present at too high or too a low concentration, the NO/H_2_O_2_ balance is disturbed, and these molecules are thus unable to induce the HR response [31]. Through examination of the NO/H_2_O_2_ ratio in *B. cinerea* plus *C. rosea* treatment group, we determined that these compounds were not in a state of equilibrium, which may explain why we did not observe a toxic reaction in this group.

We observed that the second messenger mechanism varied according to each type of treatment. The effect of induction was greater in *B. cinerea* plus *C. rosea* treatment than in the other treatment, and the induction time was also shorter. Therefore, this type of induction must be highly effective, leading to the hypothesis that *C. rosea* can induce resistance to tomato facing *B. cinerea* infection.

### Changes in expression of *MAPK* and *WRKY* in tomato leaves under *C. rosea* treatment

Studies of the early events that follow pathogen recognition have established the importance of mitogen-activated protein kinase (MAPK) cascades in plant defense signaling. Plant WRKY transcription factors are key regulatory components of plant responses to microbial infection, in addition to regulating the expression of defense-related genes. In this study, by examining the expression of MAPK and WRKY genes, we found that these genes were more highly expressed in *B. cinerea* plus *C. rosea* treatment than in the other two treatments. Meanwhile the expression levels of these genes were higher after *C. rosea* treatment than *B. cinerea* treatment. Both types of genes were abundantly expressed in a short period of time, and the expression of these genes was longer lasting and more consistent than that in the other groups. Several other studies have also shown that the reaction systems of WRKY and MAPK participate in plant resistance. MAPK cascades involving NbMKK1 control non-host resistance, including HR cell death, and WRKY33 is an important transcription factor that regulates the antagonistic relationship between defense pathway-mediated responses to *P. syringae* and necrotrophic pathogens [32], [33].

### Change in phytohormone levels in tomato leaves under *C. rosea* treatment

Jasmonic acid (JA) is a well-characterized signaling molecule in plant defense responses. Jasmonic acid (JA), salicylic acid (SA), methyl jasmonate (MeJA) and ethylene are endogenous hormones, and they play a role in maintaining the resistance of non-host plants as well as microbial interactions.

High performance liquid chromatography can be used to quickly determine the levels of a variety of endogenous plant hormones such as ABA, IAA, GA3 and ZT, as well as salicylic acid and methyl jasmonate. In this study, by determining the levels of endogenous hormones, we found that the contents of IAA and ZT were unchanged in the different treatment groups, except for *C. rosea* treatment, where these two hormones were present at high concentrations, which suggests that *C. rosea* could promote plant growth.

Rice dwarf virus (RDV)-infected rice plants exhibit a significant reduction in GA levels, and treatment of infected plants with GA_3_ restores the normal growth phenotype [34]. In the current study, tomato leaves infected with *B. cinerea* exhibited a low content of GA_3_. In the other two treatment groups, in which *C. rosea* was present, there was a high content of GA_3_, which suggests that GA_3_ associated with *C. rosea* infection can participate in the resistance against the disease pathogen.

ABA is a growth inhibitor. ABA activates stomatal closure, which acts as a barrier against bacterial infection [35], and it may be involved in the negative regulation of plant defense against various pathogens. In this study, there was a low level of ABA detected in all three treatment groups, perhaps due to bidirectional antagonism between ABA and SA, as demonstrated Yasuda. suggesting that ABA participates in an indirect manner to the fight against *B. cinerea* infection [36].

Recently, several studies have reported that disease resistance in *Arabidopsi*s is regulated by multiple signal transduction pathways in which salicylic acid (SA), jasmonic acid (JA) and ethylene (ET) function as key signaling molecules. Jasmonic acid (JA) is a well-characterized signaling molecule that functions in plant defense responses. Enyedi showed that SA levels increased dramatically in tobacco cells surrounding infection sites that were infected by *Tobacco mosaic* virus [37]. Our study shows that the three treatments caused an exponential increase in ethylene content, but *B. cinerea* treatment produced the highest value, which could be caused by the interaction between the plant and *B. cinerea*. The infection of tomato leaves by *B. cinerea* induces the biosynthesis of ethylene and increases ethylene content.

In this study, we found a high level of SA with a maximum value at 96 h in *B. cinerea* treatment compared to the control, although this value was less than that induced by the other two treatments. These results are in agreement with those of Enyedi [37], who observed a high level of SA in tobacco plant cells in the presence of tobacco mosaic virus infection. Despite the high level of SA induced by *C. rosea* treated and *B. cinerea* plus *C. rosea* treated, SA did not cause any hypersensitive reaction in these plants, for the following reasons: perhaps its concentration had not reached the levels that can cause an accumulation of hydrogen peroxide, or perhaps SA associated with *C. rosea* can play an important role in resistance to plant diseases.

JA (or MeJA) accumulates to high levels after wounding or elicitor induced plant cell in plants and cell cultures [38], [39]. In the current study, tomato leaves treated with *B. cinerea* did not show a significant change in JA content; only a slight increase was observed. Therefore, we can assume that the development of gray mold in tomato is not related to the content of JA. This result is in agreement with that of Audenaert [40]. Tomato leaves in the *C. rosea* plus *B. cinerea* treatment and *C. rosea* treatment had a high content of JA, which suggests that JA/MeJA participate in the induction process of *C. rosea*, and that the intervention or production of JA/MeJA may induce the expression of several defense-related genes in plants, such as genes encoding PAL, PR-10/chitinase, β-1, 3-glucanase and others.

Numerous studies have shown that after a plant has undergone pathogenic infection, there is an obvious increase in the release of ethylene, suggesting that the release of ethylene represents a plant defense reaction to previous pathogens, which plays an important role in plant resistance to diseases [41], [42]. Over 60 different cultivars and breeding lines of wheat exhibit increased ethylene production as a result of infection by the fungal phytopathogen *Septoria nodorum*, which is correlated with increased plant disease susceptibility [43].

The results of the current study showed that, after the inoculation of tomato leaves by *B. cinerea*, the ethylene content increased significantly. Our results were in agreement with those of [44], who observed an increase of ethylene production in dicotyledonous plants such as cabbage, pea, carrot, cucumber, carnation, and tomato infected with *Meloidogyne javanica*.

Lund demonstrated a deficiency in the production of ethylene and a significant reduction in disease symptoms in tomato mutants compared with wild type plants after the inoculation of two genotypes with virulent bacteria (*Xanthomonas campestris* pv. *vesicatoria* and *Pseudomonas syringae* pv. *tomato*) and fungi (*Fusarium oxysporum* f. sp. *lycopersici*) pathogens [45]. In our study, the tomato leaves treated with *C. rosea* and inoculated with *B. cinerea* showed a deficiency in ethylene content compared with the other two treatments. We hypothesize that the ethylene production occurs simultaneously to the progression of disease symptoms in response to *B. cinerea* and *C. rosea* infections as a biological control agent that is capable of fighting these infections. In tomato leaves treated with *C. rosea* alone, the ethylene content also increased, and these leaves also had increased levels of IAA. We propose that IAA may induce the production of ethylene in tomato leaves in the absence of infection. This finding is contrary to the results obtained by [44], who demonstrated that the production of ethylene in tomato roots infected with *M. javanica* was accelerated by IAA. The ethylene content increased significantly after the inoculation of tomato leaves by *B. cinerea*, which may have led to the formation of lesions that appeared on the leaves. In tomato leaves treated with *C. rosea* alone, the ethylene content also increased, which may have been due to the increase in IAA levels, which cause an increase in ethylene content. In leaves treated with *C. rosea* and inoculated with *B. cinerea*, the ethylene content was low, so lesion did not occur on the tomato leaves. An increase in ethylene content can activate the plant defense process, such as the production of phytoalexin and pathogenic proteins, transformation of the cell wall and so on.

### Change of translated proteins in tomato leaves under *C. rosea* treatment

Two-dimensional gel electrophoresis is one of the core technologies used in proteome research. This technique can be used to elucidate changes in the expression of proteins related to plant disease resistance. In this study, a combination of SDS-PAGE and 2-D Image Master was used to identify proteins involved in each treatment group. Through comparative analysis, we detected a total of 50 spots (two of which corresponded to novel proteins), including commonly and specifically expressed proteins, to evaluate the differences in protein profiles between the three treatment groups and the control.

We found that *B. cinerea* plus *C. rosea* treatment had a higher level of protein expression than the other two treatments. The various functions of some of the identified proteins are listed in Table 1. *B. cinerea* plus *C. rosea* treatment induced a number of newly expressed or highly expressed proteins compared to the control, such as the following: serine-type endopeptidase (spot 3); oxidoreductase/peroxidase (spot 4); ATP binding related spot 8 (enzyme); glutathione-S-transferase (spot 18,); carbonate dehydratase (spot 20); threonine endopeptidase (spots 21, 35, and 42); hydrogen ion transporting ATP synthase (spot 24); ribosomal protein (spot 28); response to biotic stimulation (spot 32); β-xylosidase (spot 37); fructose-bisphosphate aldolase (spot 39); FAD binding/NADP or NADPH binding/ferredoxin-NADP^+^ reductase (spot 40); ATP synthase CF1 alpha subunit (spot 41); associated with Ca^2+^ binding (spot 43); pathogenesis-related protein (spot 46); ribulose bisphosphate carboxylase (spot 47) and antibacterial/fungicide/pathogenesis-related protein (spot 50).

The secretome of a tobacco cell suspension culture was previously investigated using a combined proteomic and metabolomic approach. The proteins identified were mainly involved in stress defense and cell regeneration processes. Among the proteins, three putative new isoforms, e.g. β-xylosidase, chitinase, and peroxidase were identified [46]. Tai used gel-based proteomics to study the effect of stress on membrane protein expression in maize (*Zea mays* L.) [47]. In the current study, two-dimensional gel electrophoresis led to the identification of several proteins, e.g., an ATP synthase CF1 alpha subunit, GAPDH and etc.

Our results showed that treatment of tomato leaves with *C. rosea* significantly increased the activity and expression levels of proteins (Fig. 6). The two-dimensional gel electrophoresis analysis revealed new types of proteins were induced in *B. cinerea* plus *C. rosea* treatment compared with other treatments and water control, i.e., spots 37 and 41. Spot 37 corresponds to LEXYL2, and spot 41 corresponds to ATP synthase CF1 alpha subunit. LEXYL2 (β-xylosidase) acts as a hydrolase. LEXYL2(EC 3.2.1.37) is a kind of hemicellulose hydrolase responsible for the hydrolysis of β-D-xylosyl residues of xylans that are widely in plant cell wall. Previous study found that the expression of LEXYL2 in tomato leaves was induced by tomato yellow leaf curl virus (TYLCV). The expression level is 3.06-fold up-regulated in TYLCV-resistant line than in TYLCV-susceptible line [48]. For plant disease resistant, LEXYL2 could be involved in plant cell wall modification or rearrangement, or provide one signal for plant disease resistant reaction. This need be further experimentally investigated. ATP synthase CF1 alpha subunit, which was located at chloroplast, plays a role in the proton transport mechanism of ATP synthase. The activity of ATPase played an important role in promoting plant resistance to abiotic and biotic stress [49], [50]. ATP synthase CF1 alpha subunit trigger one series of resistance reaction by proton transportation and improving cytoplasmic pH or providing the signal molecule. ATP synthase CF1 alpha subunit also could facilitate increasing the energy for enhancing the resistance of plants [51]. Meanwhile, the expression of *atpA* gene was higher than that of *LEXYL2* gene after *B. cinerea* plus *C. rosea* treatment and *C. rosea* treatment. The results illustrated that *atpA* gene could play more important role than *LEXYL2*. New proteins specifically expressed in tomato leaves inoculated with *B. cinerea* and then induced by *C. rosea* may be associated with non-host resistance functions in plants, but the functions of these proteins are still being investigated. The results of real-time PCR confirmed those from two-dimensional gel electrophoresis, i.e., the presence and level of expression of two proteins, ATP and Lexyl2, at 72 h in *B. cinerea* plus *C. rosea* treatment.

### Biocontrol activities and mechanisms of *C. rosea*

Several studies have successfully demonstrated the effectiveness of the use of *C. rosea* for the biological control of several phytopathogenic fungi. *C. rosea* is a biocontrol agent that is used to combat and prevent phytopathogenic fungal attacks that involve many factors and diverse modes of action. *C. rosea* can suppress the production of spores and produce hydrolytic enzymes (e.g., protease and chitinase), which are likely to play a key role in its ability to penetrate and kill a host. Moreover, this fungus also can induce the resistance of plants [21]. In this study, *C. rosea*, and especially *C. rosea* treatment after *B. cinerea* inoculation, can induce the resistance of tomato plants according to the physiological index, key gene expression levels and protein changes. These results also indicated that *C. rosea* treatment after *B. cinerea* inoculation induces the expression of specific proteins, including LEXYL2 (β-xylosidase, spot 37) and ATP synthase CF1 alpha subunit (spot 41, Fig. 6). Overall, all changes in some physiological indexes (defense enzymes, NO, H_2_O_2_, O_2_^−^, GA3, MeJA, SA, ethylene), as well as MAPK and WRKY expression levels, showed that *C. rosea* treatment plus *B. cinerea* inoculation can induce the resistance of tomato leaves most significantly among the three treatments and the control. *C. rosea* treatment can induce the resistance of tomato leaves more strongly than *B. cinerea* inoculation alone. This phenomenon might be due to either the accumulation of resistance or the production of new effectors that induce resistance. Compared to the control, the changes and resistance of some physiological indexes between three different treatments differed, and these results illustrated that the three treatments have different mechanisms of inducing plant resistance.

In conclusion, *C. rosea* can induce the resistance of tomatoes to *B. cinerea*. Moreover, β-xylosidase and ATP synthase CF1 alpha subunit are the key proteins that function in the resistance of tomato to *B. cinerea*.
